# INTERSECTIONALITY AND QUALITATIVE HEALTH RESEARCH: POTENTIALS TO IMPACT QUALITATIVE RESEARCH

**DOI:** 10.1093/geroni/igac059.975

**Published:** 2022-12-20

**Authors:** Martina Roes, Franziska Laporte-Uribe, Jem Bhatt, Carolien Smits, Aud Johanessen, Viktoria Peters-Nehrenheim, Huerrem Tezcan-Guentekin

**Affiliations:** German Center for Neurodegenerative Diseases (DZNE), Witten, Nordrhein-Westfalen, Germany; German Center for Neurodegenerative Diseases (DZNE), Witten, Nordrhein-Westfalen, Germany; UCL, Unit for Stigma Research, London, England, United Kingdom; Pharos, Utrecht, Utrecht, Netherlands; University of South-Eastern Norway, Kongsberg, Vestfold, Norway; DZNE, Göttingen, Nordrhein-Westfalen, Germany; ASH, Berlin, Berlin, Germany

## Abstract

With a growing interest in intersectionality, critical qualitative researchers acknowledge the benefit from incorporating an intersectional lens. Incorporated intersectionality can be beneficial since it allows researchers from different disciplines to consider and account for the participant identities as multidimensional, fluid and, interdependent at each stage of the research process. In this presentation we will offer insights how qualitative research can be enhanced by incorporating an intersectional lens when analyzing the interconnectedness of numerous socially constructed identities (such as sex/gender/ethnicity/transnational families). Furthermore, we will address challenges how to conduct intersectional qualitative research (e.g. intersectional oriented interviews) and analysis (e.g. anticategorial, intracategorial and intercategorial complexity). This approach challenges on many levels, but since qualitative and interdisciplinary research is about utilizing meaning of phenomena, incorporating an intersectional lens will not only expand the methodological horizon of researchers but also stimulate ethical and critical inquiry throughout the research process.

